# Protective Effect of Mannitol on Cisplatin-Induced Nephrotoxicity: A Systematic Review and Meta-Analysis

**DOI:** 10.3389/fonc.2021.804685

**Published:** 2021-12-16

**Authors:** Songtao Li, Xiuyun He, Linjie Ruan, Ting Ye, Yulong Wen, Zhihua Song, Siying Hu, Yu Chen, Bo Peng, Shijie Li

**Affiliations:** ^1^ Department of Oncology, Hospital of Chengdu University of Traditional Chinese Medicine, Chengdu, China; ^2^ Clinical School, Chengdu University of Traditional Chinese Medicine, Chengdu, China; ^3^ Fudan University Shanghai Cancer Center, Fudan University, Shanghai, China

**Keywords:** mannitol, cisplatin, nephrotoxicity, cisplatin-induced nephrotoxicity, meta-analysis

## Abstract

**Introduction:**

Cisplatin, a chemotherapeutic drug, is widely used for the treatment of various malignant tumors with good effects. However, cisplatin-induced nephrotoxicity is a major dose-limiting factor and a significant adverse event. Mannitol is used to reduce cisplatin-induced nephrotoxicity, which is controversial. This study aimed to evaluate the efficacy and safety of a hydration regimen containing mannitol against cisplatin-induced nephrotoxicity through a meta-analysis.

**Methods:**

Potential records from PubMed, EMBASE, Cochrane Library, and ClinicalTrials that met the inclusion criteria were included from inception to May 2021. Cochrane Collaboration tools were used to assess the risk of bias in the included studies. Jadad’s and NOS scores were applied to assess the quality of randomized controlled trials (RCTs) and case-control studies. A random-effects model or fixed-effects model was used depending on the heterogeneity. Subgroup analyses were performed to evaluate the potential study characteristics. The pooled odds ratios (ORs) and 95% confidence intervals (CIs) were evaluated.

**Results:**

Four RCTs and seven case-control studies involving 4168 patients were included. Pooled results showed that mannitol use could reduce the incidence of cisplatin-induced nephrotoxicity (OR = 0.66, 95% CI [0.45–0.97], *p* = 0.03), especially reducing grade 3 nephrotoxicity events according to CTCAE 4.0 (OR = 0.37,95% CI [0.16–0.84]). Moreover, mannitol use was not significantly associated with creatinine clearance, serum creatine, and electrolyte disturbance (*p > 0.05*). Gastrointestinal cancer (OR = 0.36, 95% CI [0.15–0.83], *p* = 0.02) and urinary tract cancer (OR = 0.32,95% CI [0.14–0.73], *p* = 0.007) may be more sensitive to mannitol, although the test for overall effect was significantly different (OR = 0.66, 95% CI [0.49–0.89], *p* = 0.007). For patients with diabetes and hypertension, mannitol may worsen renal function (OR = 1.80, 95% CI [1.18–2.72], *p* = 0.006; OR = 2.19, 95% CI [1.50, 3.19], *p* < 0.0001, respectively). Mannitol may have a better protective effect when doses of mannitol were ≥ 25 g (OR = 0.58, 95% CI [0.39–0.88], *p* = 0.01) and doses of cisplatin < 75 mg/m^2^ (OR = 0.59, 95% CI [0.36–0.94], *p* = 0.03). It revealed that mannitol use was likely to cause nausea or vomiting (OR = 1.86, 95% CI [1.20–2.89], *p* = 0.006).

**Conclusion:**

Current evidence revealed that mannitol was an effective and safe drug to reduce cisplatin-induced nephrotoxicity events, especially Grade 3 events. However, it may cause more nausea/vomiting events and deteriorate renal function in patients with diabetes or hypertension. We also found that mannitol had the best effect when mannitol was ≥ 25 g in total or cisplatin was < 75 mg/m^2^. Meanwhile, mannitol may have a better effect on gastrointestinal and urinary tract cancers.

**Systematic Review Registration:**

crd. york. ac. uk/PROSPERO, CRD 42021253990

## Introduction

Cisplatin (CDDP; cis-dichlorodiammine platinum [II]) is a non-specific drug of the cell cycle with strong cytotoxicity and broad-spectrum antitumor effects; it is widely used in urogenital system tumors, malignant lymphoma, breast cancer, head and neck cancer, non-small cell lung cancer, etc. (1–3) Cisplatin-induced nephrotoxicity is one of the main reasons for the dose limitation (4–6). In fact, one-third of treated patients account for the overall incidence of cisplatin-induced nephrotoxicity (7). The primary mechanisms of cisplatin-induced nephrotoxicity are proximal tubular injury, inflammation, oxidative stress, and vascular injury in the kidneys (8). Proximal tubular injury is related to the energy-dependent uptake of cisplatin in renal cells (9) and organic cationic transporters (OCTs) in proximal renal tubules, especially OCT2 (3, 10–13). In addition, most cisplatin accumulates in the renal cortex (9, 14) and has a direct killing effect on renal epithelial cells (1, 15). *In vivo*, necrosis and apoptosis can be induced by cisplatin, regardless of dosage (16). Inflammatory cells and cytokines play essential roles in cisplatin-induced nephrotoxicity. Increased levels of IL-1, IL-6, IL-18 (17), and TNF-α (18) are associated with cisplatin-induced nephrotoxicity. Compared with TNF-α, TNFR-2 plays a more critical role in nephrotoxicity development (19). CD4+ T cells (17), macrophages (20), and mast cells (21) mediate the inflammatory process of cisplatin-induced kidney injury. Production of reactive oxygen species (ROS), accumulation of lipid peroxidation products in the kidney, and inhibition of the antioxidant system may be the main mechanisms of cisplatin-induced nephrotoxicity (22). Cisplatin may also cause mitochondrial dysfunction and increase ROS production through the damaged respiratory chain (23). Cisplatin induces acute ischemic injury, resulting in decreased renal medullary blood flow (24) and renal tubular cell damage (25). Meanwhile, cisplatin can block calcium ion channels to reduce COX-2 and prostaglandin, cause vasoconstriction, and eventually lead to aggravation of hypoxia (26). Vascular endothelial injury also plays a vital role in cisplatin-induced nephrotoxicity because it is directly toxic to endothelial cells (27). Currently, short-term rehydration is mainly used to prevent the occurrence of renal toxicity (28), but there is no evidence to prove this effect.

Nephrotoxicity is the primary dose-limiting toxicity of cisplatin (29, 30), which involves glomerular or tubular dysfunction of the kidneys (31). There are several standard methods to measure kidney function and define nephrotoxicity or acute kidney injuries (32–34), such as creatinine clearance, Common Terminology Criteria for Adverse Events (CTCAE), glomerular filtration rate, RIFLE (risk, injury, failure, loss, and end-stage), and serum creatine urinary activity of N-acetyl-beta-D-glycosaminidase (NAG). In the clinic, some different hydration protocols used during the period of cisplatin infusion, including dextrose 5%, normal saline, the electrolyte solution, and diuretics such as mannitol and furosemide (28). Some clinical trials have indicated that mannitol has a positive effect on cisplatin-induced nephrotoxicity (35, 36), but mannitol has nephroprotective effects that have not proven (37).

Mannitol (C6H14O6) is the hexacarbonyl of mannose and is widely used as an osmotic diuretic with strong dehydrating effects (38–40). Mannitol is currently used in hydration regimens to prevent the renal toxicity of cisplatin, but it remains controversial (41), mainly because mannitol itself may cause kidney damage. Mannitol can cause extensive equal-length proximal renal tubule vacuolization, strong afferent arteriole constriction, and acute renal failure (42, 43). Studies have shown that high doses of mannitol can inhibit the proliferation of renal tubular epithelial cells in a time- and dose-dependent manner (44). More importantly, mannitol is a hydroxyl radical scavenger that can reduce cisplatin-induced cytotoxicity by inhibiting oxidative stress (45). We aimed to evaluate the efficacy and safety of mannitol against cisplatin-induced nephrotoxicity through a meta-analysis to provide objective evidence for the application of mannitol in the hydration regimen of cisplatin.

## Materials and Methods

### Protocol Registration

The present study was registered in PROSPERO on June 2021 (registration No. CRD42021253990, crd.york.ac.uk/PROSPERO)

### Search Strategy

Two reviewers (Ruan and Ye) independently searched PubMed, EMBASE, Cochrane Library, and ClinicalTrials.gov independently, with no time or language restrictions. The search string was made as follows: “cisplatin” and “mannitol” and “nephrotoxicity” or “kidney injuries” or “renal injury” or “kidney failures” or “renal failures” or “renal insufficiency.”

### Study Selection

Inclusion criteria were as follows: (i) type of studies, including retrospective and prospective studies, (ii) patients with cancer (regardless of cancer type) who received cisplatin alone or in combination with other chemotherapeutic agents, (iii) studies comparing hydration regimens with and without mannitol, (iv) the primary outcomes were assessments of nephrotoxicity (SCr, eGFR, and creatinine clearance) or the incidence of AKI. Additional outcomes were electrolyte disturbance (hypomagnesemia, hypokalemia, and hypernatremia) and adverse events.

Exclusion criteria were as follows: (i) Animal experiments, review articles, commentaries, case reports, and meta-analyses; (ii) Irrelevant studies, duplicate literature, and records without valuable data.

### Data Extraction

Data extraction was performed by two investigators independently. The following information was extracted: (i) Study ID, composed of the first author’s name and publication year; (ii) Country where the study was conducted; (iii) Study subjects, number of participants, sex, age, type of cancer, and study design; (iv) Treatment regimens for the treatment and control groups, including the drugs and doses; (v) Outcomes of each study. One study had different groups of data or data at different time points, which were extracted independently, as in another study.

### Quality Assessment

Two reviewers (Wen and Song) independently performed a quality assessment of the literature. The risk of bias was assessed using the Cochrane Collaboration tools. Given no restriction on study types, RCTs were evaluated using Jadad’s scale (0–5) (46), while non-RCTs were evaluated using the Newcastle-Ottawa Scale(0–9) (47). Jadad’s scale assessed the quality of the study based on three criteria: (i) randomization (0–2); (ii) double blinding (0–2); and (iii) withdrawals and dropouts (0–1). A final score of three or above was considered high quality; otherwise, it was considered to be of low quality. NOS judged the study regarding three aspects: selection of the study groups, comparability of the groups, and exposure of study groups. The final score was higher and the quality was more reliable. Any discrepancies during the assessment was resolved by consensus or discussion with the corresponding author.

### Statistical Analysis

All statistical analyses were conducted using the Review Manager software (version 5.3) and STATA SE (version 16.0). Since both prospective trials (RCTs) and retrospective trials (case-control) were included in this analysis, odds ratios (ORs) with 95% confidence intervals (CIs) were used to evaluate the incidence of kidney injury between treatment regimens with and without mannitol, as well as for other dichotomous data. In contrast, continuous data were reported as mean differences with 95% CIs to summarize the results. The pooled effect depended on the heterogeneity of the included literature, which was assessed using a Q test and an I^2^ test (48). A random-effects model was used when I^2^ > 50% and P < 0.05; otherwise, a fixed-effects model was adopted. Subgroup analysis was conducted to review the incidence of kidney injury according to the grade of nephrotoxicity, cancer type, dose of cisplatin, dose of mannitol, and demographic characteristics.

## Results

### Literature Search Outcome

**Figure 1:**
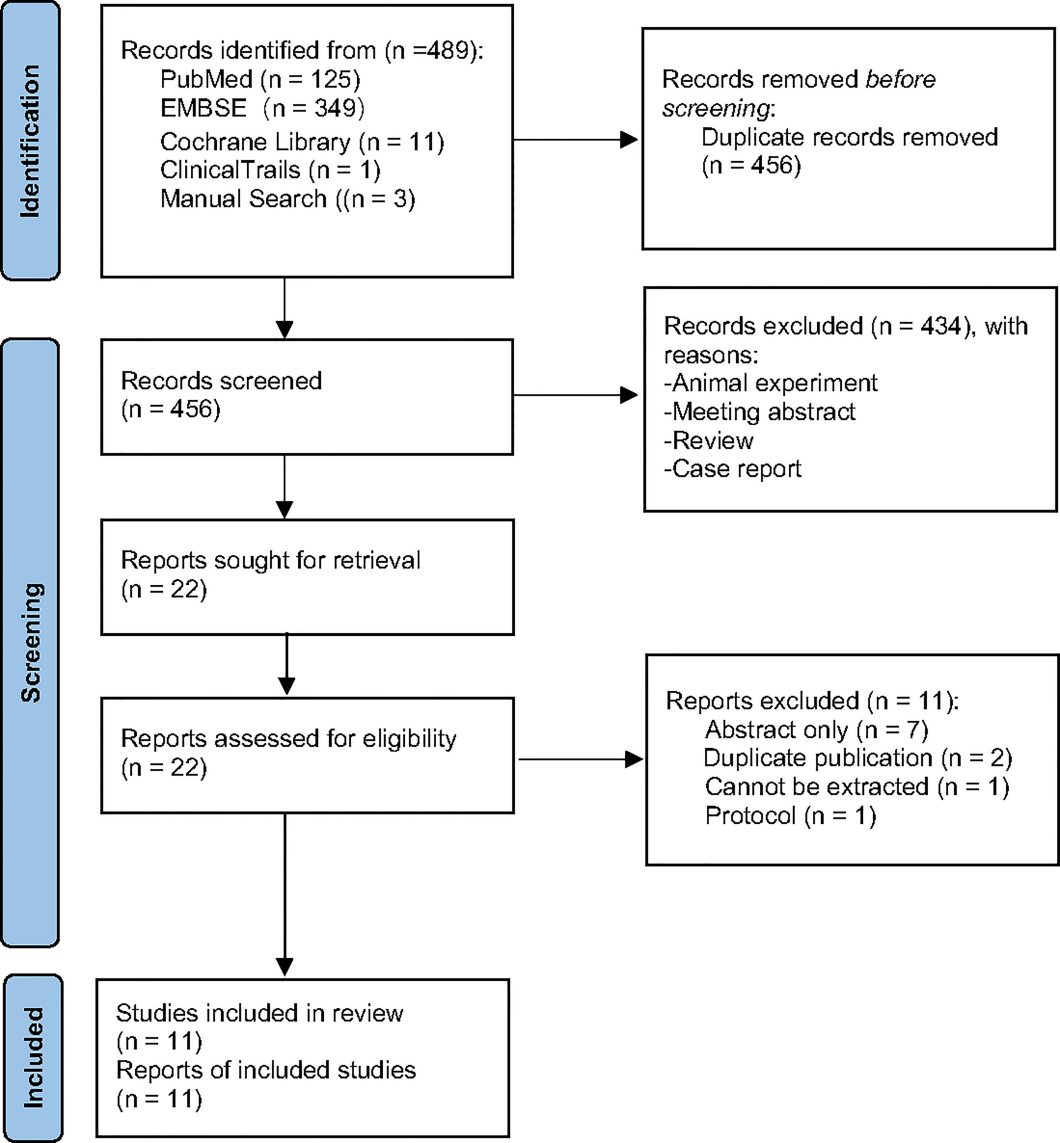
PRISMA 2020 flow diagram.

A total of 489 potential records were collected from PubMed, EMBASE, Cochrane Library, and ClinicalTrials.gov from inception to May 2021. After duplicates were removed, 434 articles were excluded through screening of the titles and abstracts, as they were mainly animal experiments, abstracts, reviews, and case reports. Twenty-two full-text records were carefully assessed for eligibility; 11 articles were excluded because of abstract only (n = 7), duplicate publication (n = 2), data loss (n = 1), and protocol (n = 1). Eventually, 11 studies were included for further analysis (**Figure 1**).

### Literature Characteristics

A total of 11 studies involving 4168 patients from the USA, Canada, and Japan were included in the present meta-analysis. One thousand three hundred ninety-five patients received mannitol before or after cisplatin treatment. Four studies were RCTs (49–52), while seven were case-control studies (12, 35, 53–57). To better and more comprehensively evaluate the effect of mannitol on cisplatin-induced kidney injury, we divided some articles into different studies without affecting the integrity of the data. *Makimoto 2020* (50) recorded data of patients at first and all courses of treatment, respectively, and it was divided into two studies: *Makimoto 2020 (First)* for data at first course and *Makimoto 2020(Entire)* for data over the entire course*. Morgan 2014* (35) integrally recorded data according to the different doses of cisplatin; therefore, we regarded it as two different studies: *Morgan 2014 (100 mg)* for data at 100 mg/m^2^ of cisplatin and *Morgan 2014 (30 mg)* for data at 30 mg/m^2^ of cisplatin*. Santoso (2003)* compared mannitol with saline and furosemide. Therefore, it was divided into *Santoso 200 3(Furosemide)* for data compared to furosemide and *Santoso 2003 (saline)* for data compared to saline. While *Bégin 2020* (12) classified cancer types, different data were used for subgroup analyses (**Table 1**).

**Table 1:** Basic characteristics of the included studies.

Study ID	Type	Design	Ration	Cancer types	Cisplatin (mg/m^2^)	Mannitol	Controls	Outcomes	Adverse events
Bégin (12)	Retro	Case-control	Canada	HN(17.7%)/Lu(19%)/Gyn(13.7%)/GI(10.4%)/Gen(10.2%)/Lym(11.1%)/Germ(5.8%)/Others(12.1%)	< 75./≥ 75	12.5g/25g	Saline	HR	NO
Dhillon (44)	Retro	Case-control	Canada	Gen(20.5%)/Gyn(18.9%)Lu(36.5%)/GI(15.6%)/Breast(2.3%)Lym(4.6%)HN(1.6%)	≥ 50	12.5g/37.5g	Saline	OR	NO
Dimery (40)	Pro	RCT	USA	HN(84%/)Skin(6%)/Other(42)	≥ 75	25g	Saline	OS, ION	YES
Leu (45)	Retro	Case-control	USA	Lu(44.5%)/GI(20.1%)/HN(16.3%)/Cervical(15.2%)/Others(3.9%)	>40	12.5 g	Saline	ION,CrCL,TTR	NO
Mach (46)	Retro	Case-control	USA	Cervical cancer	40	24g	Furosemide	Scr,CrCL,Mg,HR	NO
Makimoto (41)	Pro	RCT	Japan	NSCLC	75-80	UN	Furosemide	ION,ScrOS	YES
McKibbin (47)	Retro	Case-control	USA	HN	100	12.5g	Saline	ION, CrCL, INED	NO
Morgan (48)	Retro	Case-control	USA	HN(97.2%) Others(3.8%)	30/100	25g	Saline	ION, OR	YES
Muhyl (42)	Pro	RCT	USA	Malignant melanoma	100	35.5g	Saline	INO, OS	YES
Santoso (43)	Pro	RCT	USA	Gyn	50-75	50g	Saline/furosemide	CrCl, Scr,24h-CrCl	NO
Williams (49)	Retro	Case-control	USA	HN(28.4%)/Lu(26.2%)/Gyn(24.6%)/Gen(10.5%)/Others (10.3%)	≥40	25/12.5g	Saline	ION	NO

Retro, Retrospective study; Pro, prospective study; HN, head and Neck; Lu, Lung; Gyn, Gynecologic; GI, gastrointestinal; Gen, Genitourinary; Lym, Lymphoma; Germ, Germ cell; CrCL, Creatinine clearance; ION, Incidence of nephrotoxicity; Scr, Serum creatinine; TTR, Time to recover; Mg, Serum magnesium; IOED, Incidence of electrolyte disturbances; NSCLC, non-small cell lung cancer. HR, Hazard Ratio; OR, Odd Ratio; OS, Overall Survival; UN, Unknown.

### Literatures Quality Assessment

**Figure 2:**
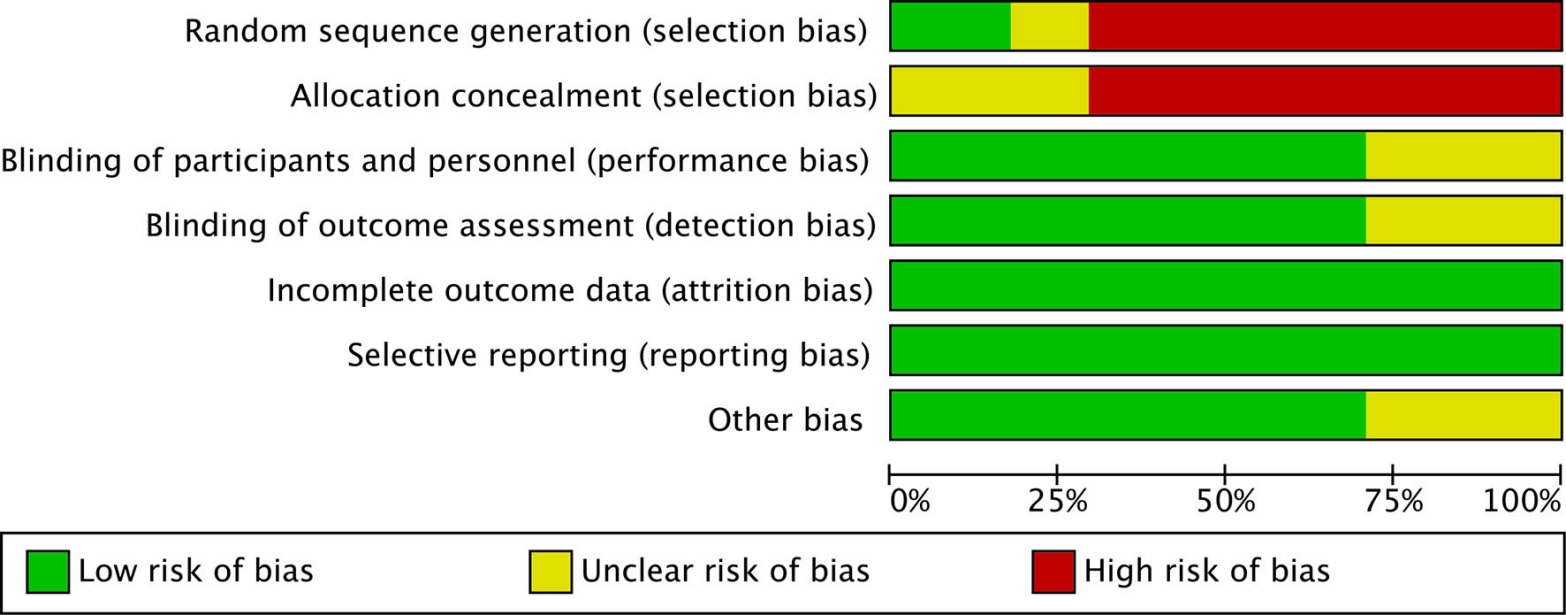
Results of literature quality risk of bias summary.

**Figure 3:**
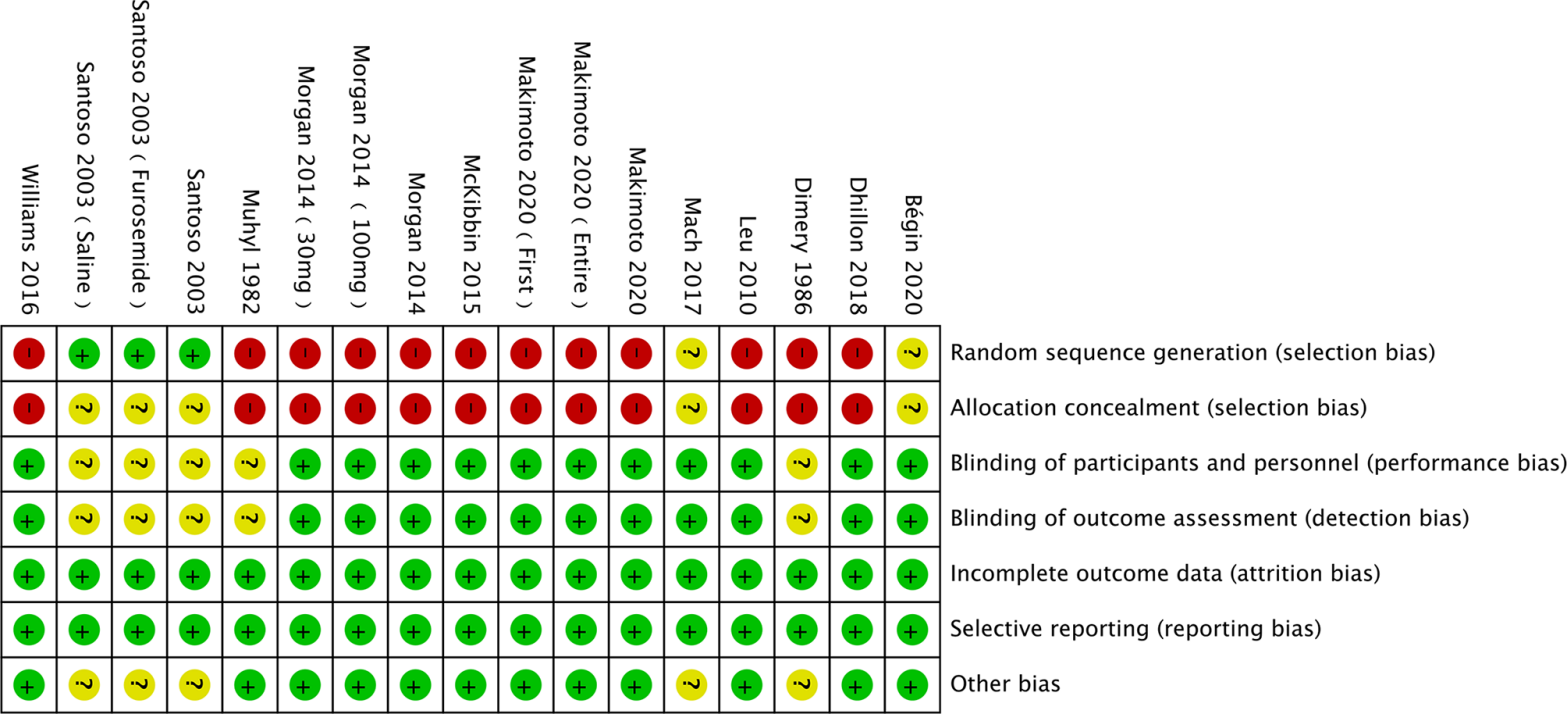
Results of literature quality risk of bias graph.

The risk of bias was assessed using the Cochrane Collaboration tool (**Figures 2**, **3**). Random sequence and allocation concealment were generally missing, mainly because 7 out of 11 pieces of literature were case–control studies. Jadad’s score (**Table 2**) and NOS (**Table 3**) were used to evaluate the quality of the included RCTs and case–control studies, respectively. The Jadad score ranged from 2 to 3 out of 5. One trial (52) was regarded as high quality ((3 ≥ points), while all of the case-control studies were considered high quality (range 7 to 8 points).

**Table 2:** Results of quality assessment using the Jadad Score (5-points) for RCTs.

Study	Randomization			Double blinding			Withdrawals & Dropouts		Scores
	No	Sketchy	Detailed	No	Sketchy	Detailed	No	Yes	
Dimery (40)	1			0			1		2
Makimoto (41)	1			0			1		2
Muhyl (42)	1			0			1		2
Santoso (52)	2			0			1		3

**Table 3:** Results of quality assessment using the Newcastle-Ottawa Scale (NOS) for case–control studies.

Study	Selection				Comparability	Exposure			Scores
	Adequate definition of cases	Representativeness of the cases	Selection of controls	Definition of controls	Controls for important factor^#^	Ascertainment of exposure	Non-response rate	Same method of ascertainment for cases and controls	
Bégin (12)	★	★		★	★★	★	★	★	8
Mach (46)	★	★		★	★★	★	★	★	8
Dhillon (44)	★	★		★	★	★	★	★	7
McKibbin (47)	★	★		★	★★	★	★	★	8
Morgan (48)	★	★		★	★	★	★	★	7
Williams (49)	★	★		★	★	★	★	★	7
Leu (45)	★	★		★	★	★	★	★	7

^#^A maximum of 2 stars can be allotted in this category, one for mannitol, another for other controlled factors.

### Incidence of Cisplatin-Induced Nephrotoxicity (ICN)

**Figure 4:**
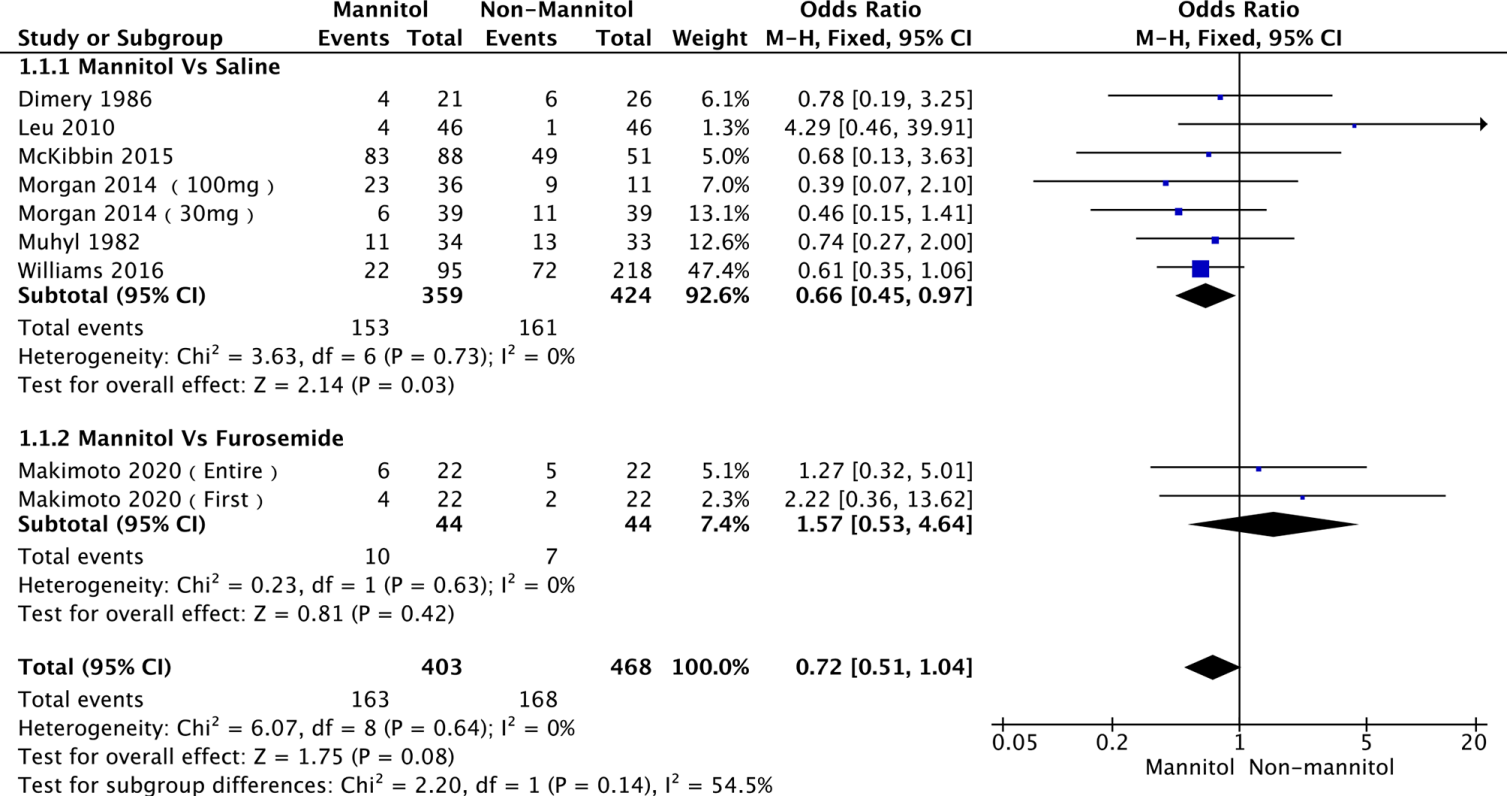
Forest plot of odds ratios (OR) for incidence of cisplatin-induced nephrotoxicity (ICN).

A total of 7 (35, 49–51, 54, 56, 57) out of 11 studies analyzed the effect of mannitol on the incidence of cisplatin-induced nephrotoxicity (ICN). Two subgroups were divided according to the control treatment. Six studies (49, 51, 54, 56, 57) were into “saline,” while *Makimoto 2020* (50) [including *Makimoto 2020 (entire)* and *Makimoto 2020 (first)]* were into “furosemide.” There was no heterogeneity across the six saline studies (*p* = 0.73, I^2^ = 0%) and furosemide studies (*p =* 0.63, I^2^ = 0%). Therefore, a fixed-effects model was used for pooled analysis. In the pooled meta-analysis, ICN was significantly different between mannitol and saline (OR = 0.66; 95% CI [0.45-0.97], *p* = 0.03). In the furosemide subgroup, ICN did not differ between mannitol and furosemide (OR = 1.57;95% CI [0.53–4.64], *p* = 0.42). The analysis results are presented in **Figure 4**.

### Incidence of Cisplatin-Induced Nephrotoxicity (Grade)

**Figure 5:**
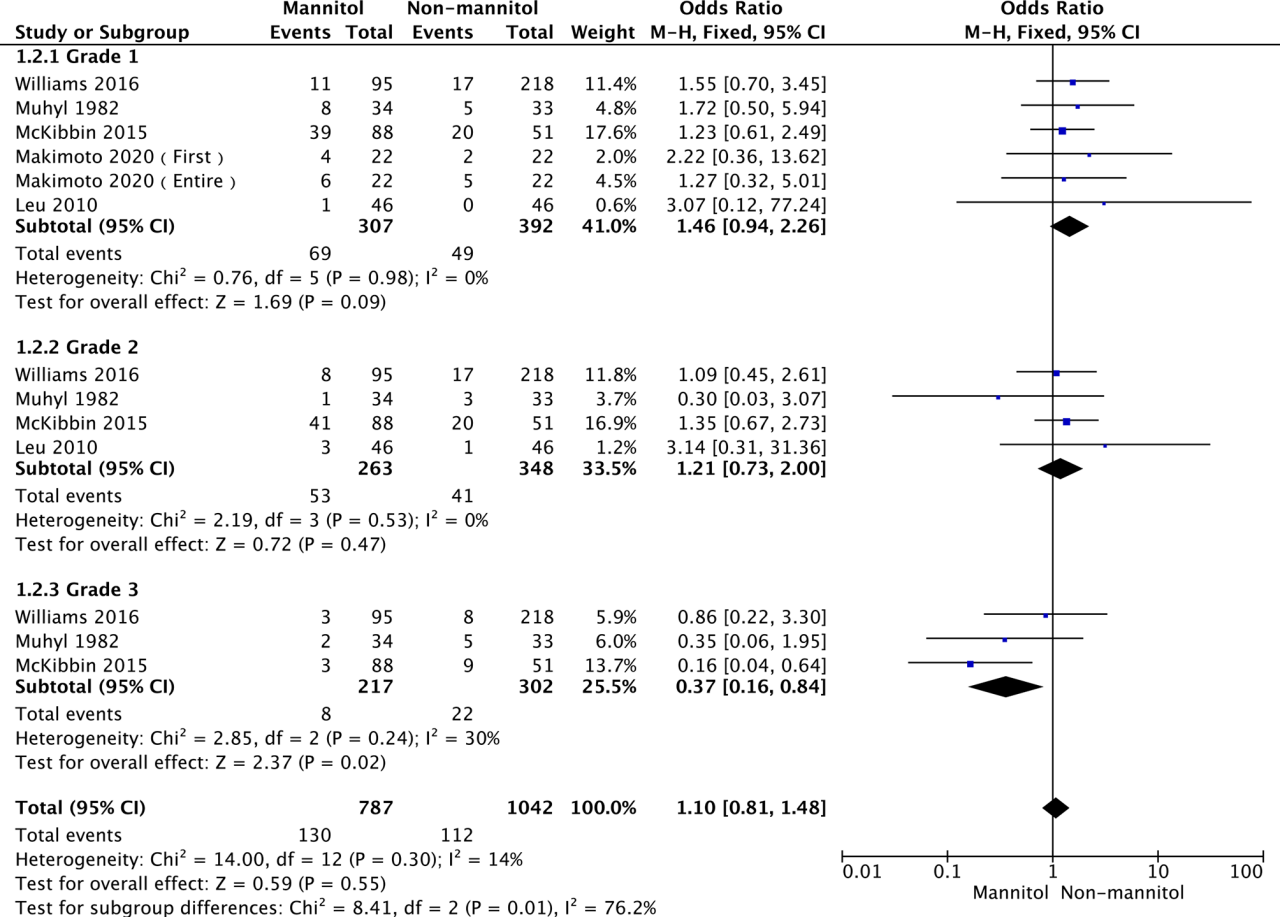
Forest plot of odds ratios (OR) for incidence of cisplatin-induced nephrotoxicity (Grade).

According to Common Terminology Criteria for Adverse Events v4.0 (CTCAE 4.0), Acute kidney injury was classified into 5 grades (1 to 5, 5 was worst). Subgroup analysis was performed in three groups according to renal failure grade. In this analysis, five studies (50, 54, 56–58) reported the incidence of cisplatin-induced nephrotoxicity (ICN) to Grade 1, four (51, 54, 56, 57) reported ICN as Grade 2, and three (51, 56, 57) reported ICN as Grade 3.The overall heterogeneity was not significant (*p* =0.30, I^2^ = 14%). Thus, a fixed-effects model was used. There were no statistically significant differences in grade 1 (OR = 1.46, 95% CI [0.94–2.26], *p* = 0.09) and grade 2 (OR = 1.21,95% CI [0.73–2.00], *p* = 0.47). Regarding grade 3, ICN was remarkably different between mannitol and non-mannitol (OR = 0.37, 95% CI [0.16–0.84], *p* =0.02). Details are shown in **Figure 5**.

### Creatinine Clearance and Serum Creatinine

**Figure 6:**
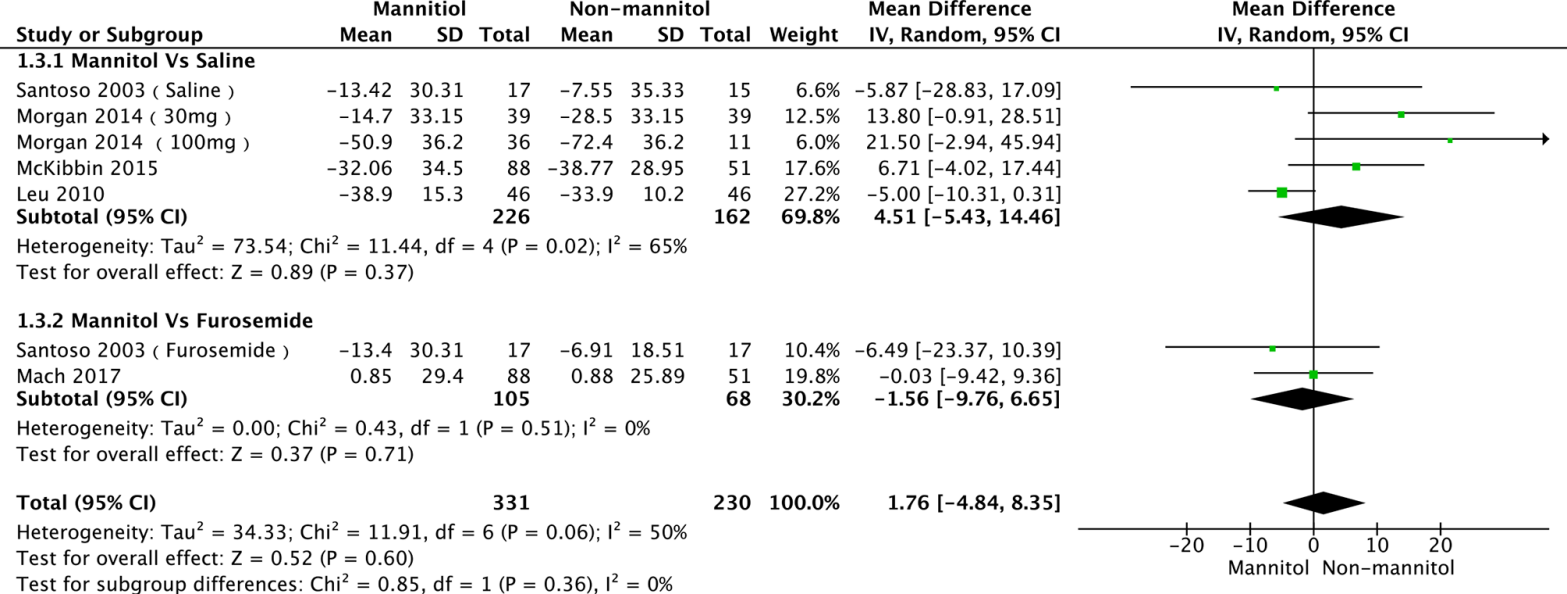
Forest plot of mean difference (MD) for Creatinine Clearance (CrCl).

**Figure 7:**
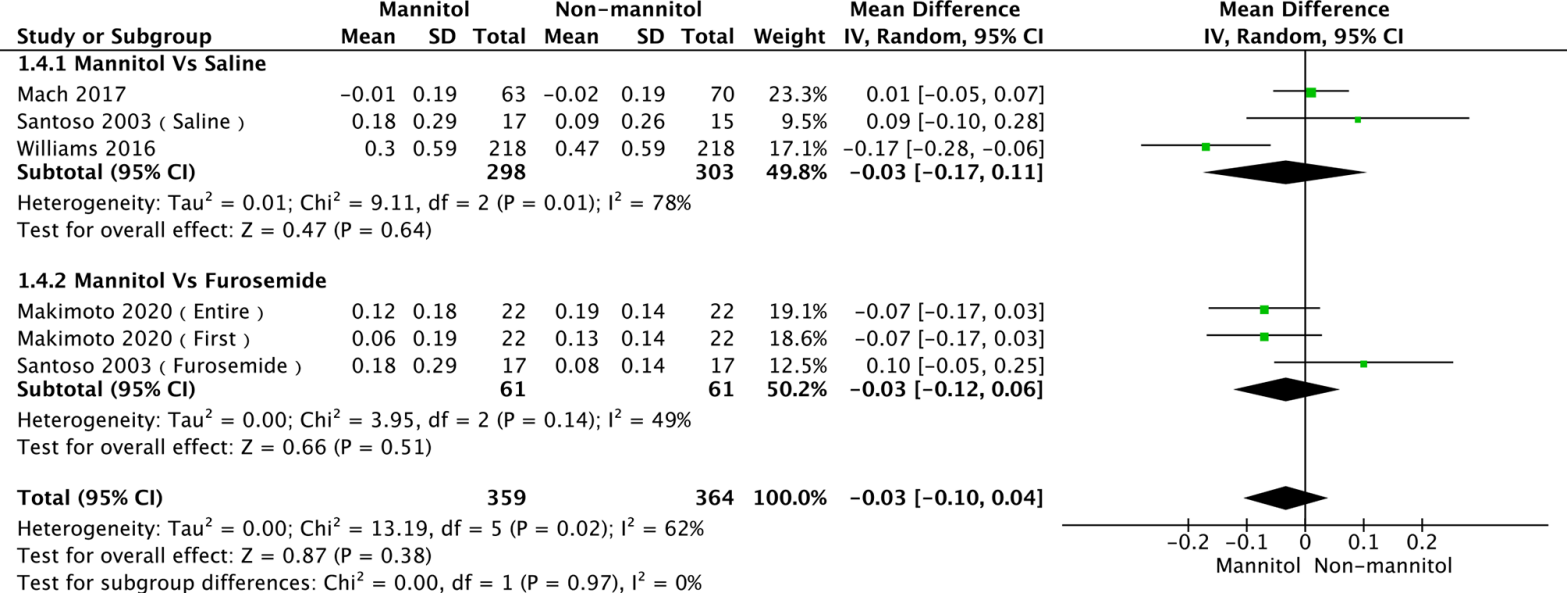
Forest plot of mean difference (MD) for Serum Creatinine (Scr).

A total of seven studies (35, 52, 55, 56, 58) were evaluated in creatinine clearance analysis, divided into saline and furosemide subgroups, depending on the control method. *Leu 2021* was the main reason for the heterogeneity in the saline group. There was no heterogeneity (*p* = 0.36, I^2^ = 6%). The test results of saline (mean difference [MD] = 4.51, 95%CI [-5.43–14.46], *p* = 0.37), furosemide (MD = -1.56, 95% CI [-9.76–6.65], *p* = 0.71), and overall groups (MD = 1.76, 95% CI [-4.84–8.35], *p* =0.60) are presented in **Figure 6**. Regarding the meta-analysis of serum creatinine, four studies (50, 52, 55, 57) were initially included. *Williams 2016* was the primary reason for the high heterogeneity (with it, *p* = 0.44, I^2^ = 0%). There were no differences between mannitol and saline (*p* = 0.64) or furosemide (*p* = 0.51) (**Figure 7**)

### Electrolyte Disturbance

**Figure 8:**
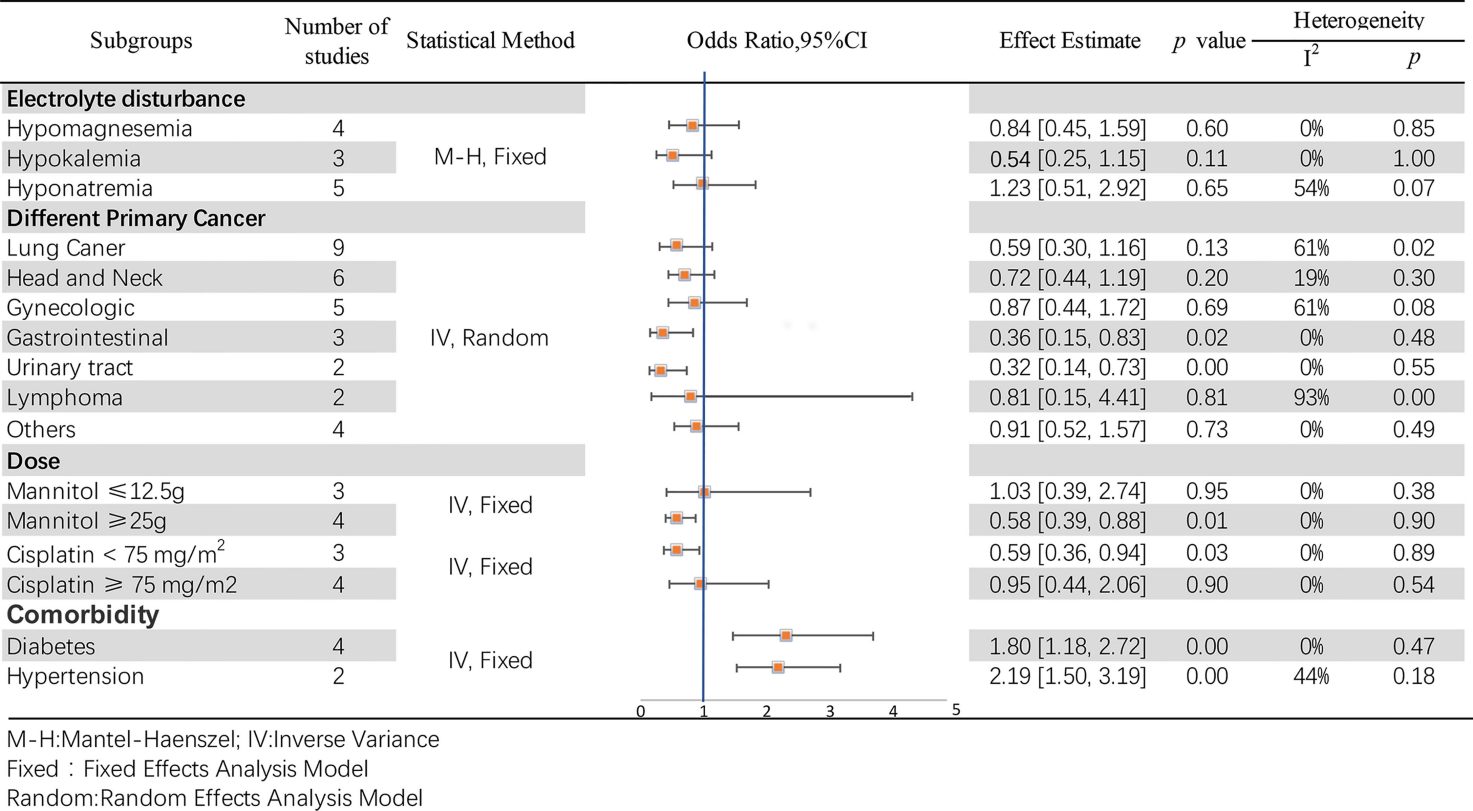
Subgroup analyses of the association between mannitol and cisplatin-induced nephrotoxicity.

Hypomagnesemia, hypokalemia, and hyponatremia were the three most common electrolyte disorders. In the hypomagnesemia subgroup, four studies (35, 49, 56) were included, while there was no incidence of hypomagnesemia in *McKibbin 2015*, and no statistically significant differences were found between them (OR = 0.84, 95% CI [0.45–1.59], *p* = 0.60). Three studies were analyzed in the hypokalemia subgroup; no significant differences were observed between *McKibbin 2015*, *Morgan 2014 (100 mg)*, and *Morgan 2014 (30 mg)* (OR = 0.54, 95% CI [0.25–1.15], *p* = 0.11). Although the hypomagnesemia and hypokalemia groups showed no heterogeneity (*p* = 0.85, I^2^ = 0%; p =1.00, I^2^ = 0%, respectively), the hyponatremia subgroup was moderately heterogeneous (*p* = 0.07, I^2^ = 54%; however, the test effect was not significant (OR = 1.23, 95% CI [0.51–2.92], *p* = 0.65). A pool analysis is presented in **Figure 8**.

### Primary Cancer Types

In this pool analysis, the effects of mannitol on cisplatin-induced nephrotoxicity were observed in different primary cancer types, including lung (12, 50, 53, 56, 57), head and neck (12, 35, 53, 57), gynecologic (12, 53, 57), gastrointestinal (12, 53), urinary tract (12, 57), lymphoma (12, 53) and other cancer types (12, 51, 53, 57). The results reveal statistical differences in gastrointestinal (OR = 0.36, 95% CI [0.15–0.83], *p* = 0.02) and urinary tract (OR = 0.32, 95% CI [0.14–0.73], p =0.007) cancers, but otherwise in other subgroups (*p >*0.05). Additionally, the test for overall effect was significantly different (OR = 0.66, 95% CI [0.49–0.89], *p* =0.007). Details at **Figure 8**.

### Doses of Mannitol and Cisplatin

According to the common clinical usage of mannitol and cisplatin, subgroups were established as mannitol dose ≤ 12.5 g or ≥ 25 g, and cisplatin dose < 75 mg/m^2^ or ≥ 75 mg/m^2^, respectively. Differences were observed regardless of the dose of mannitol (OR = 0.64,95% CI [0.44–0.93], *p* = 0.02). Particularly, it may have a better protective effect (OR = 0.58, 95% CI [0.39–0.88], *p* = 0.01) when mannitol ≥ 25 g. Details are shown in **Figure 8**. There were remarkable differences when the cisplatin dose was < 75 mg/m^2^ (OR = 0.59, 95% CI [0.36–0.94], *p* = 0.03). In contrast, no differences were observed when the cisplatin dose was ≥ 75 mg/m^2^ (OR = 0.95, 95% CI [0.44–2.06], *p* = 0.90). For these two groups, the effect is Z = 1.94 (*p* = 0.05), which is statistically different. The results are presented in **Figure 8**.

### Comorbidity

Diabetes and hypertension were the two most common comorbidities in the included studies. *Dhillon 2018* (53), *Mach 2017* (55), *McKibbin 2015* (56) and *Morgan 2014* (35) reported a correlation between diabetes and mannitol-cisplatin-induced nephrotoxicity. These four studies showed high heterogeneity (*p* = 0.05, I^2^ = 62%; OR = 1.80, 95%CI [1.18–2.72], *p* = 0.006), mainly due to *Morgan 2014* (35)(without it, *p* = 0.47,I^2^ = 0%; OR = 2.32, 95% CI [1.46, 3.68], *p* = 0.0003). Regarding the correlation between hypertension and mannitol-cisplatin-induced nephrotoxicity, Mach 2017 (55) and Morgan 2014 (35) showed remarkable differences (OR = 2.19, 95% CI [1.50, 3.19], *p* < 0.0001), and no heterogeneity (*p* = 0.18, I^2^ = 44%). Details are shown in **Figure 8**.

### Adverse Event

**Figure 9:**
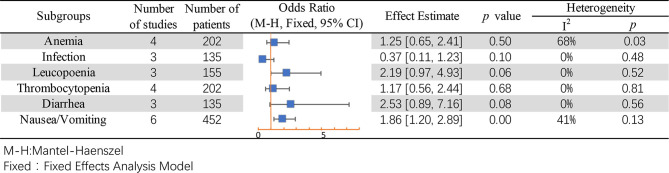
Adverse events of mannitol use in cisplatin-induced nephrotoxicity.

Subgroup analysis was performed for common adverse events, including anemia (49–51), infection (49, 50), leukopenia (50, 51), thrombocytopenia (49–51), diarrhea (49, 50), and nausea or vomiting (35, 49–51). Except for the result of nausea/vomiting being different between mannitol and non-mannitol (OR = 1.86, 95% CI [1.20–2.89], *p* = 0.006), there were no differences in other subgroups (**Figure 9**).

### Sensitivity Analysis and Publication Bias

**Figure 10:**
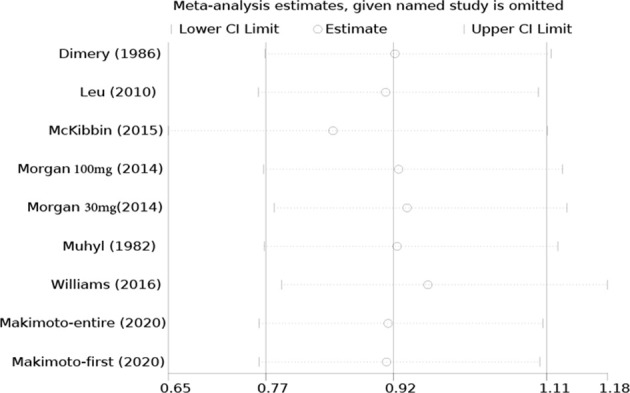
Sensitivity analysis of the effect of individual studies on the pooled ORs for mannitol and cisplatin-induced nephrotoxicity.

**Figure 11:**
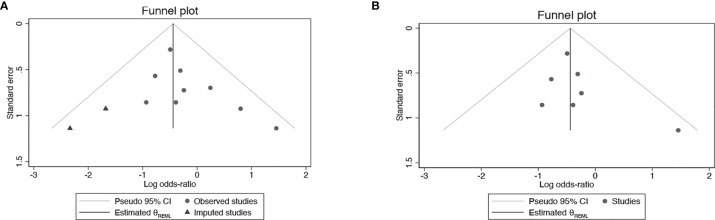
Publication bias with trim and fill **(A)** Mannitol Vs. Non-mannitol **(B)** Mannitol Vs. Saline.

All studies were successively omitted to assess the robustness of the pooled results. Begg’s and Egger’s tests were combined to evaluate whether a publication bias existed. As shown in **Figure 10**, the significance of the recalculated ORs did not change when any study was omitted. This indicated that the correlation between mannitol use and the reduction in the incidence of cisplatin-induced nephrotoxicity was robustly significant. Of note, the results of Begg’s test and Egger’s test showed no sign of publication bias for this study, with p-values of 0.076 and 0.554, respectively. Hence, we performed a sensitivity analysis using the trim and fill method to evaluate the effect of potential publication bias. Strangely, Begg’s test and Egger’s test showed that there was no publication bias, while the trim and fill method suggested that there was a lack of literature. Thus, two hypothetical negative studies were added to diminish the asymmetry of the funnel plot; although the result changed, the conclusion was not affected (9 observed studies [log OR = -0.336, 95% CI (-0.706–0.034)], 9 observed +2 imputed studies [log OR = -0.440, 95% CI (-0.798 – -0.082)]. We considered that saline and furosemide were included in the control group in the included literature. Then, we only performed the trim and filling method for the saline group and found no publication bias (log OR = -0.437, 95% CI [-0.831 – -0.044]) (**Figure 11**).

## Discussion

Cisplatin is a very effective and widely used anti-tumor drug that carries a significant risk of nephrotoxicity. Cisplatin-induced nephrotoxicity is a primary adverse effect, leading to limited cisplatin use. Mannitol has been used to reduce this risk. To date, studies evaluating the role of mannitol in preventing cisplatin-induced nephrotoxicity are controversial. Therefore, we conducted this meta-analysis.

Although there is a recognized causal relationship between cisplatin and kidney injury, there is no accurate prediction of the incidence of cisplatin-related nephrotoxicity. Moreover, we aimed to evaluate the protective effects of mannitol on cisplatin-induced nephrotoxicity rather than its therapeutic effects. Therefore, in this meta-analysis, RCTs (49–52) and case-control studies (12, 35, 53–57) were included in order to obtain a better and more comprehensive assessment of the impact of mannitol on cisplatin-induced nephrotoxicity.

Meanwhile, to ensure the quality of this meta-analysis, we used the Jadad scale and NOS scale to assess the quality of included RCTs and case–control studies, respectively. Generally, all of the included RCTs lacked mention of double-blinding. The quality assessment results of RCTs were four (49–51) as low quality (score at 2) and one (52) as high quality (score at 3). Seven case controls were regarded as high-quality studies according to the results of NOS (range, 7–8), mainly due to the bias of controls for essential factors.

We found that mannitol significantly reduced the incidence of cisplatin-induced nephrotoxicity, especially when compared with normal saline (*p = 0.03*), while there was no statistically significant difference compared with furosemide (*p = 0.42*). These results may be associated with the speed of cisplatin metabolism *via* the kidneys for mannitol and furosemide. However, furosemide may cause hearing loss and other adverse events (59, 60). Nevertheless, further studies and more pieces of literature are needed to confirm whether furosemide and mannitol have different protective effects on cisplatin-induced nephrotoxicity. Interestingly, in a previous study, it was found that there was no difference in the effect of mannitol on kidney injury. However, in a meta-analysis, the protective and preventive effects of mannitol on kidney injury were confirmed by expanding the sample quantity. Subsequently, subgroup analyses were performed to assess whether mannitol differed in the protective effects of different grades of kidney injury. According to the Common Terminology Criteria for Adverse Events V4.0 (CTCAE 4.0), potential cisplatin-induced nephrotoxicity events were classified as grade 1–5. There was no significant difference between grade 1 and grade 2 renal injury. Although there was significant heterogeneity in grade 2, using a leave-one-out procedure at a time, the results were stable. More importantly, mannitol had a significant protective effect on grade 3 renal injury (*p <* 0.0001). This cloud was associated with a comparison of mannitol with normal saline in the study included in grade 3. Subsequently, we excluded studies that used furosemide as a control agent. We found that there were no differences in grade 1 (*p = 0.12*) and grade 2 (*p = 0.84*). Further studies and further research are needed to confirm these results.

The heterogeneity of the subgroup of creatinine clearance in Leu 2021 was due to the fact that patients received an unusual dose of cisplatin (cisplatin, 40 mg/m^2^) and mannitol (12.5 g). While 89 patients received 25 g of mannitol and 6 received 12.5 g in Williams 2016, the different doses of mannitol may be the reason for the heterogeneity of the subgroup of serum creatinine. The results show that mannitol can reduce the incidence of cisplatin-induced nephrotoxicity by contrasted with saline; however, mannitol cannot decrease the creatinine clearance and serum creatinine level. This result strongly indicates that mannitol contributes to cisplatin-induced nephrotoxicity, not by accelerating glomerular filtration (61), but by other mechanisms, such as mitophagy (62) and oxidative stress (63). Three major pathways that regulate mitophagy, including PINK1/PARK2 pathway (64–66), DRP-1dependent pathway (67), and HIF-1α/BNIP3/BCEN-1 pathway (68), play protect roles in cisplatin-induced AKI. However, mannitol does not activate HIF-1 (69), but it can increase DRP-1 in mitochondria (70) to inhibit mitochondrial dysfunction. Meanwhile, mannitol as an antioxidant (71–73) can protect kidney function by inhibiting oxidative stress. Thus, we assume mannitol could reduce the incidence of cisplatin-induced nephrotoxicity, especially for grade 3, which may be associated with mitophagy and anti-oxidative stress, which should be verified by further study.

Diuretics contribute to electrolyte disorders, often promoting the excretion of electrolytes into urine by the kidneys. In this meta-analysis, the effect of mannitol on electrolytes was specifically analyzed, and it was found that mannitol did not exacerbate electrolyte disorders, including hypomagnesemia, hypokalemia, and hyponatremia.

A subgroup analysis was performed to verify whether mannitol differed in renal protective function among different cancer types. Furthermore, we found that the cancers originated from the gastrointestinal and urinary tract and that mannitol may have a more remarkable protective effect. Studies show that p53, the well-known tumor suppressor, has a preventive and therapeutic effect on cisplatin-induced kidney injury (74–76). Nevertheless, mannitol has a remarkable protective effect on the gastrointestinal and urinary tract, which may be associated with the different expression of p53 (77) and also the same origination embryologically (78, 79). Furthermore, KRAS (Kirsten rat sarcoma viral oncogene) is frequently mutated in gastrointestinal cancers, plays an important and protective role on renal function (80). For urinary tract cancer, Eph/ephrin signaling regulates the development of cancer growth and kidney injury (81). However, more and further reaches should be verified.

Cisplatin is a dose-limiting chemotherapeutic drug, primarily due to kidney injury. This explains why mannitol has a positive protective effect when cisplatin dose < 75 mg/m^2^, while no significant protective effect was observed for doses ≥ 75 mg/m^2^. The protective effect of mannitol on renal function has been confirmed in several previous studies, and we found that mannitol doses ≥ 25 g may have a better effect. However, there was no difference in efficacy between half and total doses of mannitol in the treatment of cerebral hemorrhage (82).

We found that mannitol may have opposite effects on the comorbidity. Morgan 2014 led to significant heterogeneity, although it did not influence the results, which included two different groups: cisplatin 30 mg and 100 mg. Nevertheless, log-regression was conducted regardless of the cisplatin dose. The results indicated that mannitol might aggravate kidney injury in patients with diabetes or hypertension. Hypertension (83) and diabetes (84) facilitate glomerular structural injury and progressive loss of renal function. Mannitol could worsen renal function when it had already been damaged (42), but this requires further research.

We also evaluated the safety of the use of mannitol. According to the reports in the included literature, adverse events were mainly found in the hematopoietic and digestive systems. Among these, nausea and vomiting were the main adverse reactions to mannitol. This may be related to the impact of mannitol on intestinal permeability (85).

Sensitivity analysis and publication bias were conducted to assess the robustness of the pooled results; Begg’s test and Egger’s test showed no publication bias, but an inadequate number of studies could not be used to draw the Begg’s plot or Egger’s plot. Then, we continued to carry out the trim and fill method to verify the potential publication bias, and the conclusion was not affected by the addition of two hypothetical negative studies.

However, our study has limitations. First, the number of eligible studies for this meta-analysis and subgroup analyses were limited. Since we did not extract individual patient data from the included studies, subgroup analyses were conducted according to study-level data. Second, although the overall heterogeneity was weak in this meta-analysis, the subgroup analysis revealed that the amount of mannitol, concentration of cisplatin, and type of primary tumor might contribute to heterogeneity. In addition, chronic disease conditions, such as hypertension and diabetes, may deteriorate the protective effect of mannitol (35, 53, 55, 56). More studies and further studies will testify to the effect of mannitol on the protection of kidney function. Third, due to the small number of articles included, we could not assess publication bias in this meta-analysis.

In conclusion, the results of the meta-analysis based on evidence from RCT and case-control studies revealed that mannitol was an effective and safe drug to reduce cisplatin-induced nephrotoxicity events. Although mannitol cannot directly reduce the clearance of serum and endogenous creatinine, it can reduce the incidence of renal injury events, especially in grade 3 kidney injury events. Mannitol generally does not enhance the risk of hypomagnesemia, hypokalemia, and hyponatremia. However, this may cause more nausea/vomiting events. We found that mannitol had the best effect when mannitol was ≥ 25 g in total, and cisplatin was < 75 mg/m^2^. Meanwhile, the effect of mannitol on gastrointestinal and urinary tract cancers may be better.

## Data Availability Statement

The original contributions presented in the study are included in the article/supplementary material. Further inquiries can be directed to the corresponding authors.

## Author Contributions

STL designed the study, performed the literature search and screening, performed the data analyses, and drafted the manuscript. XH guided this study and revised the manuscript. LR and TY extracted the literature and data, analyzed the extracted data, and took part in the writing of this manuscript. YW, ZS, and SH assisted in the design of the study, performed the literature search and screening, assisted in the data analyses, and YC took part in the writing of the manuscript. SJL, and BP supervised and guided the study. All authors approved the final version of this study.

## Conflict of Interest

The authors declare that the research was conducted in the absence of any commercial or financial relationships that could be construed as a potential conflict of interest.

## Publisher’s Note

All claims expressed in this article are solely those of the authors and do not necessarily represent those of their affiliated organizations, or those of the publisher, the editors and the reviewers. Any product that may be evaluated in this article, or claim that may be made by its manufacturer, is not guaranteed or endorsed by the publisher.
